# Plants strike back: Kiwellin proteins as a modular toolbox for plant defense mechanisms

**DOI:** 10.1080/19420889.2019.1586049

**Published:** 2019-02-26

**Authors:** Gert Bange, Florian Altegoer

**Affiliations:** Center for Synthetic Microbiology (SYNMIKRO) and Department of Chemistry, Philipps-University Marburg, Marburg, Germany

**Keywords:** Pathogen-related proteins, *Ustilago maydis*, chorismate, kiwellin

## Abstract

Plants have to cope with numerous stresses in nature to avoid damage or cell death. We recently reported a class of plant defense proteins termed kiwellins that were initially found in kiwifruit and shown to be causative to human food allergies. While kiwifruits among other domestic fruits always contain high amounts of kiwellin protein, available transcriptome data indicate an up-regulation of kiwellin genes upon pathogen contact in various other plants. In the case of an interaction between maize plant and the smut fungus Ustilago maydis, we could identify one kiwellin (termed: ZmKWL1) highly up-regulated in response to pathogen attack. During infection of the maize plant, U. maydis secretes numerous effector proteins that modulate the host. Among 20 predicted kiwellins, ZmKWL1 specifically inhibits the metabolic activity of the secreted fungal chorismate mutase 1 (Cmu1). We expand the current knowledge on kiwellins and describe a novel class of versatile plant defense proteins.

The extracellular space (ECS) describes the intercellular compartment between cells including cell wall components. In plants, the fluid also known as apoplast is a continuous space spanning the entire plant that enables various functions from solute transport to intercellular signaling and metabolic regulation[1].

It is likely that this large compartment is rich in proteins that are secreted and either soluble or attached to the cell-wall. Several studies have shown that the diversity of proteins in this large space is indeed high but while most of them are associated with the cell wall (and therefore cell wall-related processes), there is only a few abundant soluble proteins[1]. Among those proteins, several “pathogenesis-related” (PR) proteins are by far the most abundant [1–3].

Due to their abundance, many studies have focused on some of these proteins and more precisely their impact on human health by e.g. causing allergic reactions. In kiwifruit, several PR-proteins have been identified and characterized, namely the cysteine protease actinidain, thaumatin-like proteins, kirola and also a protein of unknown function termed kiwellin [4–6]. It appears obvious that abundant proteins identified in fruits might be causative to food allergies as toll-like receptors (TLR) usually recognize ample proteins with low stringency as e.g. in the case of bacterial flagellin[7]. While the function of actinidin [5] and thaumatin-like proteins is reasonably well understood[8], information on the functions of e.g. kirola and kiwellin is scarce. Therefore, one of the important questions to answer is the reason for their abundance and the relevance in planta.

We recently reported the interaction of the major kiwellin of *Zea mays* (ZmKWL1) with the secreted *Ustilago maydis* chorismate mutase (Cmu1) (Figure 1). Using a combination of X-ray crystallography and biochemical methods we could determine the mechanistic basis for this interaction and show that ZmKWL1 greatly reduces the activity of Cmu1[9]. Although this is the first mechanistic evidence for antifungal activity of kiwellin proteins, it is just a small piece in a large puzzle. The maize genome encodes 20 kiwellin paralogs and although others are not highly expressed under the experimental conditions tested, it is likely that they confer increased resistance to other effector proteins.

To further elucidate their function, kiwellin proteins have already been compared to other proteins with known structures according to their tertiary structure. The analysis resulted in the identification of several unrelated proteins mainly involved in carbohydrate binding or hydrolysis (expansins, cerato-platanins, endoglucanases) and PR4-like proteins, namely carwin and barwin, which have been shown to partially exhibit ribonuclease activity (Figure 1) [10–13]. To date, carbohydrate hydrolysis or ribonuclease activity could not be shown for kiwellin proteins. However, one might speculate that kiwellin proteins need to be armed by foreign effector molecules to render them active. This scenario would also explain the high abundance of these proteins in kiwifruit, where kiwellin proteins might serve as a first line of defense against pathogens. Cleavage of kiwellins into kissper and KiTH *in vitro* [6] might allow to further diversify a cellular answer by distinct roles of the two polypeptides (Figure 1). Future research needs to clarify whether Kiwellin-effector complexes exhibit activities that have been shown for other double-psi-barrel proteins earlier.

**Figure 1.**
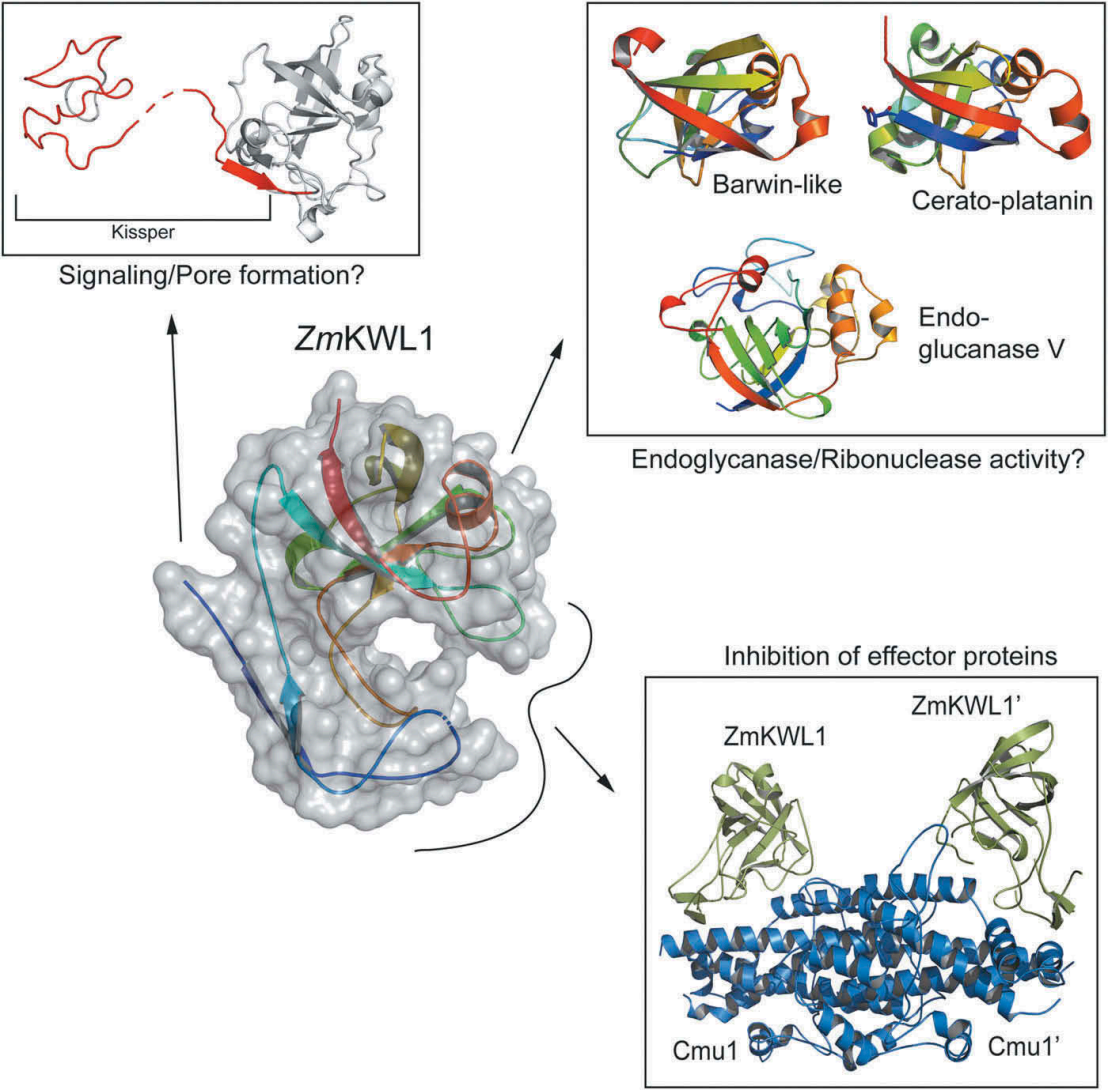
Versatility of kiwellin proteins. Kiwellin proteins have been described as modular, with the kissper peptide being cleaved. The double-ɸ -barrel has a high structural homology to barwin-like, cerato-platanin and endoglucanase V proteins while the loops protruding from the barrel mediate the interaction with effector proteins in the case of Cmu1.

Last but not least, the diversity of kiwellin proteins with more than 40 paralogs in some species might allow plants to cope with a broad-range of pathogens. Evidence that kiwellins are not solely expressed in the presence of fungi exists in e.g. potato and tomato [2,14], where at least one kiwellin is highly upregulated in the presence of an oomycete and whiteflies, respectively. Evaluation of transcriptomic data of plants grown infected by bacterial pathogens will most likely also show an upregulation of kiwellin encoding genes, making kiwellins a versatile class of plant defense proteins.
